# Intestinal Microbiota Modulation by Fecal Microbiota Transplantation in Nonalcoholic Fatty Liver Disease

**DOI:** 10.3390/biomedicines13040779

**Published:** 2025-03-23

**Authors:** Goran Hauser, Indira Benjak Horvat, Mirjana Rajilić-Stojanović, Irena Krznarić-Zrnić, Michail Kukla, Vedrana Aljinović-Vučić, Ivana Mikolašević

**Affiliations:** 1 Department of Gastroenterology, Clinical Hospital Center Rijeka, 51000 Rijeka, Croatia; goran.hauser@uniri.hr (G.H.); ikrznariczrnic@yahoo.co.uk (I.K.-Z.); ivana.mikolasevic@uniri.hr (I.M.); 2 Faculty of Medicine, University of Rijeka, 51000 Rijeka, Croatia; vedrana.aljinovic@gmail.com; 3 County Hospital Varaždin, 42000 Varaždin, Croatia; 4 Department of Biochemical Engineering & Biotechnology, Faculty of Technology and Metallurgy, University of Belgrade, 11000 Belgrade, Serbia; rajilic78@yahoo.com; 5 Department of Internal Medicine and Geriatrics, Jagiellonian University Medical College, 31-121 Cracow, Poland; kuklamich@poczta.onet.pl; 6 Department of Endoscopy, University Hospital in Cracow, 30-688 Cracow, Poland; 7 1st Infectious Diseases Ward, Gromkowski Regional Specialist Hospital, Wroclaw, 5 Koszarowa St., 50-149 Wroclaw, Poland; 8 Medical Affairs Department, Jadran Galenski Laboratorij d.d., 51000 Rijeka, Croatia

**Keywords:** nonalcoholic fatty liver disease, gut–liver axis, gut microbiota, NAFLD pathogenesis, fecal microbiota transplantation

## Abstract

Numerous factors are involved in the pathogenesis of nonalcoholic fatty liver disease (NAFLD), which are responsible for its development and progression as an independent entity, but also thanks to their simultaneous action. This is explained by the hypothesis of multiple parallel hits. These factors are insulin resistance, lipid metabolism alteration, oxidative stress, endoplasmic reticulum stress, inflammatory cytokine liberation, gut microbiota dysbiosis or gut–liver axis activation. This is a systematic review which has an aim to show the connection between intestinal microbiota and the role of its disbalance in the development of NAFLD. The gut microbiota is made from a wide spectrum of microorganisms that has a systemic impact on human health, with a well-documented role in digestion, energy metabolism, the stimulation of the immune system, synthesis of essential nutrients, etc. It has been shown that dysbiosis is associated with all three stages of chronic liver disease. Thus, the modulation of the gut microbiota has attracted research interest as a novel therapeutic approach for the management of NAFLD patients. The modification of microbiota can be achieved by substantial diet modification and the application of probiotics or prebiotics, while the most radical effects are observed by fecal microbiota transplantation (FMT). Given the results of FMT in the context of metabolic syndrome (MetS) and NAFLD in animal models and scarce pilot studies on humans, FMT seems to be a promising treatment option that could reverse intestinal dysbiosis and thereby influence the course of NAFLD.

## 1. Introduction

NAFLD (nonalcoholic fatty liver disease) represents a range of diseases from non-alcoholic fatty liver to nonalcoholic steatohepatitis (NASH), with the possible development of fibrosis and cirrhosis as the final stage that can lead to the development of hepatocellular liver cancer (HCC). A certain proportion of patients with NAFLD can potentially progress to HCC even if they do not have liver cirrhosis. Fatty liver disease is defined by excessive fat accumulation in the liver and can be a consequence of alcoholic and nonalcoholic etiologies [1]. A very important fact about NAFLD is that besides the obese population, it also affects lean people, and can lead to cryptogenic liver disease [2]. Nowadays, NAFLD is the most common cause of chronic liver disease. Given its high prevalence among adults, but increasingly among children, NAFLD is becoming a global health and economic problem that can lead to life-threatening medical conditions and diseases and could become the most common indication for liver transplantation in the next 5–10 years [3].

The global prevalence of NAFLD is approximately 30% [3,4] and is usually higher among males (40%) than females (26%) [4]. The global prevalence of NAFLD is predicted to keep growing by 2030 if current trends remain unchanged, which makes it a global health problem that requires a prompt and effective solution [4].

Nowadays, effective treatments for some chronic liver diseases are available, but treatment options for NAFLD remain inadequate. The standard of care in the case of NASH mainly involves changes in lifestyles, such as diet and physical activity, especially walking [5]. Taking that into account, the exploration of novel therapeutic approaches would be extremely important.

NAFLD is closely related to metabolic syndrome (MetS) and its individual components. Due to the increase in the prevalence of obesity, type 2 diabetes mellitus (T2DM) and MetS, the prevalence of NAFLD is also increasing. The strongest risk factors for the development of NAFLD and NASH are MetS and its individual components: obesity, especially central obesity; T2DM; dyslipidemia; and hypertension. The results of one study shows that 80% of patients with NAFLD were diagnosed with obesity and dyslipidemia, 50% with hypertension, 33% with non-insulin-dependent diabetes and 45% with a low HDL-cholesterol concentration [6]. The association of NAFLD with obesity and MetS implies that NAFLD will be more common in the next 10 years due to the global epidemic of obesity and MetS. Today the pathogenesis of NAFLD is explained by the multiple parallel hit hypothesis, according to which there are many factors involved in NAFLD development and progressions and all of them can act together simultaneously. These factors are insulin resistance, dyslipidemia, oxidative stress, endoplasmic reticulum stress, higher levels of inflammatory cytokines, intestinal dysbiosis or gut–liver axis activation [5,6,7,8,9].

The term “gut microbiota” is used to refer to a wide spectrum of microorganisms that have a role in digestion and the synthesis of vitamins as well as in the stimulation of the immune system and the decreasing of pathogens by competing for nutrients [10]. Due to this, many authors consider the gut microbiota as a “metabolic organ” or a “forgotten organ” [5,11]. In healthy adults, the gut microbiota is mostly compromised of bacteria that belong to the phyla Firmicutes, Actinobacteria and Bacteroidetes [12]. Gut microbiota homeostasis can be affected by various environmental factors including drugs (primarily antibiotics), alcohol consumption and dietary habits [13].

“Dysbiosis” is the term that describes the imbalance between protective and harmful microbes typically associated with a pathogenic condition. A major part of intestinal dysbiosis is linked to changes in gut microbiota homeostasis, especially a decrease in its variety, loss of beneficial microbiota, or the overgrowth of harmful microbiota [14]. Gut dysbiosis was shown in all three stages of chronic liver disease (CLD): steatosis; inflammation; and fibrosis. Specific changes in the gut microbiota have been reported in CLD patients suffering from NAFLD, but also from alcoholic liver disease [15,16].

The aim of this systematic review is to show the connection between the intestinal microbiota and the role of its disbalance in the development of NAFLD. We considered articles published in English in the last 30 years on PubMed/MEDLINE. The terms used were “NAFLD” and “microbiota” and “FMT”, “MAFLD” and “FMT”, “NAFLD” and “probiotics”, “gut–liver axis”. We investigated the possibility of modulating intestinal microbiota in patients with NAFLD through fecal microbiota transplantation (FMT). This represents a promising option in the treatment of NAFLD, which is regarded as an increasing global health issue, for which currently there are very few therapeutic options.

GUT–LIVER AXIS

Many articles describe the connection between the microbiota and other organs and organic systems through an axis that represents a series of complex relationships and interactions based on the signals that originate from a genetic field, environment, or even diet. Under the normal circumstances, bacteria are prevented from entering the bloodstream and translocating to places outside the intestinal lumen thanks to the epithelial barrier, as well as vascular and immunological actions that are taken to prevent translocation [10]. In the context of the epithelial barrier, different proteins called “tight junctions“ play a main role as they connect intestinal epithelial cells. On the other hand, there are two more locally acting defense mechanisms against invading microorganisms: IgA secreted by local plasma cells and vascular structures, which are important for the activation of Kupffers cells. IgA is released by B lymphocytes and forms a complex with bacteria, the result of which is the secretion of intestinal mucus. The function is multiple: it prevents bacteria from adhering to the intestinal mucosa and neutralizes toxins produced by bacteria located intraluminally. Furthermore, IgA has the ability to regulate the composition of the microbiota [17]. In addition to the above-mentioned defense mechanisms related to the intestines, the liver has its own defense mechanism—a bile mixture composed of bile acid salts, IgA, antimicrobial peptides and bicarbonates. Among the key liver products responsible for the liver–gut interaction are bile acids. The result is the induction of the production of antimicrobial peptides, as well as regulating host immunity through the induction of inflammatory genes, which results in the modulation of innate and adaptive immunity [18]. Bile acids can also play a key role in the control of the composition of the gut microbiota directly by causing the degradation of its membrane proteins [19]. Indirectly, bile acid exerts its effect by binding to the farnesoid X receptor (FXR), whose activation has been proven to be responsible for the prevention of bacterial overgrowth in the ileum by maintaining the appropriate concentration of bile acids [20]. Chenodeoxycholic acid binds to the highest extent to FXR. Its stimulation is reflected in the composition of bile acids, but it also has the ability to prevent inflammation in the liver, lipogenesis, and fibrosis, which are the features of NAFLD [21]. The main route through which the microbiota acts on the liver is enterohepatic circulation [22]. It is well established that the microbiota modulates the composition of the total bile acid pool by producing secondary bile acids. The example of the microbiota’s impact on bile acids (BAs) is the process of the deconjugation of tauro- and glyco-conjugated bile salts, which is catalyzed by the microbial bile salt hydrolase (BSH). It is known that this process occurs in the small intestine supported by all major gut microbiota phyla, including Firmicutes, Bacteroidetes, Actinobacteria and Proteobacteria [23]. The most abundant secondary BAs in humans are lithocolic acid and bacteria from the genus Clostridium are primarily important for its formation. According to all the above, we can conclude that the microbiota and the liver achieve a mutually beneficial effect through bidirectional communication via the gut–liver axis.

In a state of dysbiosis, due to changes in the composition of the intestinal microbiota, such as in NAFLD, but also in other diseases that affect the liver, the microbiota and its metabolites travel via the portal vein, causing an inflammatory reaction of the liver, which can ultimately, in some cases, result in necrosis. A predisposing factor for the translocation of the bacteria or with microbial products or cell components such as bacterial DNA or lipopolysaccharide (LPS) from Gram-negative bacteria into the bloodstream is the increased permeability of the intestinal wall, probably caused by the pathological change in the composition of the gut microbiota [24]. It is considered that liver inflammation can be induced by some of the translocated microbial products that bind to the specific pathogen recognition receptors in the guts (e.g., toll-like receptors, TLRs), thereby promoting the progression of chronic liver disease. It is presumed that the intestinal microbiota has a leading role in maintaining gut–liver axis homeostasis and in the pathogenesis of liver diseases [25]. A more detailed role of the microbiota in the development of NAFLD is explained in the next section of this article, but we can conclude that the modulation of the gut microbiota (i.e., the gut–liver axis) has attracted research interest, paving the way for novel therapeutic approaches for the management of patients with NAFLD.

## 2. Gut Microbiota Role in Metabolic Syndrome Pathogenesis

As mentioned earlier, NAFLD is closely related to MetS; thus we will first discuss the role of the gut microbiota in the context of MetS. MetS is a cluster of disorders, defined by insulin resistance (IR), arterial hypertension, dyslipidemia, increased abdominal girth and T2DM [26]. The central phenomena underlining MetS are insulin resistance and abdominal adiposity. In the case of insulin resistance, the anabolic function of insulin is disabled and the process of the inhibition of lipolysis and hepatic gluconeogenesis is disturbed. The result is an increase in circulating free fatty acids and this consequently leads to dyslipidemia related to insulin resistance [27]. Furthermore, insulin has a vasodilating effect and contributes to the regulation of blood pressure, but in the state of insulin resistance, this important regulatory function is lost and hypertension develops along with the synergistic vasoconstriction effect of the increased concentration of free fatty acid [28]. Another contributing factor to the development of metabolic syndrome is a chronic proinflammatory state characterized by an elevated concentration of tumor necrosis factor-α and IL-6 [29]. As adipose tissue is a major source of reactive oxygen species production resulting in oxidative stress and mitochondrial DNA damage, it consequently leads to the production of proinflammatory cytokines [30].

Numerous data connect the inflammatory state in MetS with gut barrier dysfunction [31]. The dysfunction of the gut barrier integrity means that the gut tissue integrity is compromised and facilitates the leakage of microbial components and/or microbes into the systemic circulation [32]. An impaired gut barrier function can occur as a malfunction of any of the gut barrier components and may lead to microbial translocation. In the context of MetS, the lymphatic system and the portal vein are the main routes for endotoxins and bacteria transportation [33]. This condition is considered as metabolic endotoxemia and can be triggered by the presence of bacterial LPS in circulation [34]. According to the earlier data, the systemic presence of microbial components can lead to the condition where inflammatory pathways are chronically activated [35]. Both local and systemic low-grade inflammation and IR are characterized by a macrophage influx activated by bacterial components that migrate to target organs. Considering that dysbiosis is the main prerequisite for the development of endotoxemia, the change in the composition of the intestinal microbiota in people with metabolic syndrome enables the chronic inflammatory process in the body to subside [35]. The composition of the microbiota in people with MetS is significantly altered compared to the healthy population (Table 1). Tomas et al. reported that a high-fat diet in mice resulted in a significantly increased abundance of the phyla Firmicutes, Proteobacteria, as well as a lower abundance of Verrucomicrobia and Bacteroidetes [36]. Some microbiota products like propionate are strongly connected to the reduction of inflammation and oxidative stress and serve as a satiety-enhancing factor [37], which would mean that in obese people, the concentration of bacterial species that dominantly produce propionate is lower. On the other hand, a study by Dalby et al. reported that metabolic endotoxemia does not link a high-fat diet to obesity [38]. Thus, further investigations are needed.

People with T2D have specific profiles of the gut microbiota, as well as in other conditions within MetS (Table 1). The number of bacteria that produce butyrate is reduced, and the number of opportunistic bacteria that disrupt the intestinal barrier, such as *Bacteroides caccae*, *Clostridium hathewayi*, *Clostridium symbiosum* and *Eggerthella Escherichia coli*, is increased [39]. Some bacterial species could be considered as a biomarker of TD2, as it was proven that *Lactobacillus* is positively correlated with fasting blood glucose (FBG) and glycosylated hemoglobin (HbA1c). On the other hand, *Clostridium* is negatively associated with HbA1c, C peptide and insulin [40].

While it has been widely recognized that the gut microbiota can contribute to the development of IR, proof has been recently generated showing that the microbiota of predisposed individuals can produce an insulin antagonist—imidazol propionate. Starting from amino acid histidine, several intestinal bacteria can produce imidazole propionate, and this microbial metabolite could indirectly activate the mechanistic target of rapamycin complex 1 (mTORC1). Interestingly, it has been shown that T2DM patients have an increased level of serum imidazole propionate and activated mTORC1 in the liver [41]. Another important mechanism when it comes to insulin sensitivity and obesity is mediated through nuclear peroxisome proliferator-activated receptors (PPARs). PPAR gamma is widely expressed in muscle, liver and adipose tissue. It is considered that through the regulation of metabolic genes’ activation of PPARg, hyperlipidemia is attenuated. Furthermore, it has been found that macrophages highly express PPARg [42]. In the context of MetS, it seems that IR and inflammation are closely connected. In addition, the accumulation of lipids in the vessel walls and chronic low-grade inflammation are proven to be intertwined. This is the reason why atherosclerosis, chronic low-grade inflammation and lipid accumulation are the main features of MetS [42].

There are some human studies that have suggested that microbiota translocation to the bloodstream and target organs [42], such as the blood vessel wall in atherosclerosis [43], the liver in NAFLD [44] and the mesenteric adipose tissue in inflammatory bowel disease [45], is very likely. Finally, the presence and activity of specific gut microbes could be associated with the development of MetS. For example, malevolent microbes such as *Prevotella copri* and *Bacteroides vulgatus* potentially contribute to IR, while *Akkermansia municiphila* and *Faecalibacterium prausnitzii* are beneficial species associated with increased insulin sensitivity [42,46,47]. A Gram-negative, mucin-degrading bacterium, *A. muciniphila*, positively correlates with a healthier metabolic status in overweight and diabetic mice and humans [48,49]. The safety of *A. muciniphila* consumption has been shown in a proof-of-concept study on humans in which supplementation with 1010 *A. muciniphila* pasteurized cells was more effective than the treatment with live cells and led to the significant improvement in insulin sensitivity, a reduction in insulinemia and a reduction in the total plasma cholesterol and the level of liver inflammation markers [50]. The role of individual species and strains in the pathogenesis of individual components of the metabolic syndrome remains to be clarified, as there are still conflicting views based on different research results regarding the possible influence on the modification of the composition of the microbiota and the extraction of potential benefits from it.

**Table 1:** Gut microbiota profile in metabolic syndrome feature.

Metabolic Syndrome Features	Gut Microbiota Profile (Abundance)	
	↑	↓
Obesity	*Firmicutes*/*Bacteroidetes* [51]	*Akkermansia* [50]
	*Lactobacillus reuteri* [52]	*Bifidobacteria* [53]
		*Lactobacillus paracasei* [54]
		*Lactobacillus gasseri* [52]
Type 2 diabetes	*Ruminococcus* [55]	*Bacteroides* [56]
	*Fusobacterium* [57]	*Faecalibacterium* [58]
	*Blautia* [59]	*Bifidobacterium*
		*Akkermansia* [60]
		*Roseburia* [55]
Dyslipidemia	*E. coli* [61]	*Akkermansia* [62]
	*Enterobacter* [61]	*Bacteroides*
		*Roseburia*
		*Faecalibacterium*

## 3. Gut Microbiota Role in NAFLD Pathogenesis

**Figure 1:**
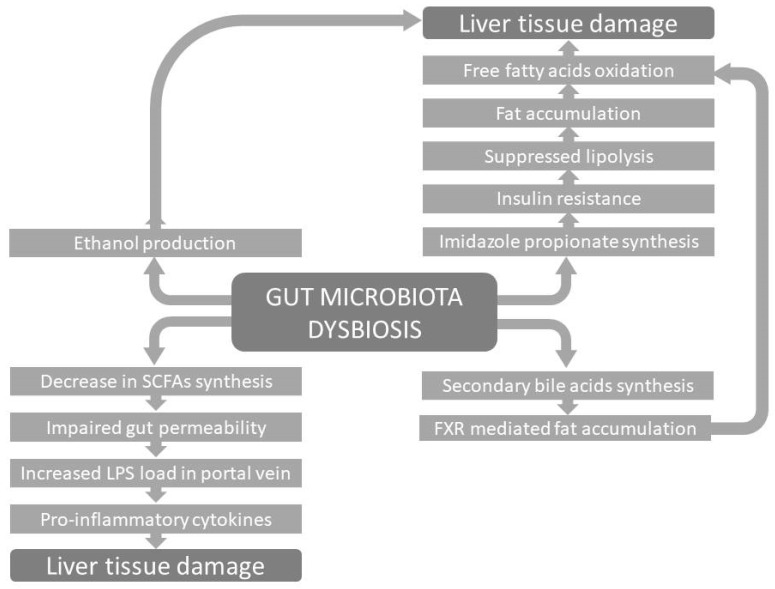
Graphical representation of the major metabolic routes by which gut microbiota dysbiosis contributes to the development of chronic liver diseases.

As was previously mentioned, NAFLD is very often connected to one or more conditions within metabolic syndrome. Due to circumstances that change the composition of the microbiota, the ability of symbiotic bacteria to maintain their function of preserving metabolic homeostasis through the secretion of important metabolites is lost. One of the most obvious direct links between the change in the microbiota composition in patients with NAFLD and the promotion of lipogenesis, as well as the deposition of lipids in the liver, is the reduced production of SCFA [63] (Figure 1). Some other known mechanisms for NAFLD development due to microbiome changes are the decreased bioavailability of choline and, on the other hand, the increased level of trimethylamine [63].

IR induced by the gut microbiota is an important mechanism by which microbiota contributes to the development of metabolic disorders, including NAFLD.

Free fatty acids (FFAs) originate from two sources: the first is the lipolysis of adipose tissue, and the second is lipogenesis from glucose and fructose. In the liver, FFAs are transformed into triglycerides that are later secreted into the blood in *very-low-density lipoprotein* (VLDL) particles or stored in hepatocytes. When the liver’s capacity to store and transform FFAs is exceeded, and this can occur in patients with IR (insulin is responsible for suppressing lipolysis), the excessive FFAs are transformed into lipotoxic substances, responsible for oxidative stress, the injury of the endoplasmic reticulum and the activation of inflammasomes and apoptotic mechanisms. All the aforementioned processes associated with excessive FFAs in the liver lead to the development of chronic inflammatory processes in the liver [46,48]. An excess of FFAs accumulated in the liver leads to more oxidative stress, thus enhancing the process described above [64,65,66,67,68].

Mediated by the nuclear receptor farnesoid X receptor (FXR), bile acids (BAs) are another class of molecules important in microbiota–host communication that could have an impact on MetS development [47,49,69]. BA binds with several receptors, such as *Takeda- G- protein receptor-5* (TGR5), FXR, *constitutive androstane receptor* (CAR), *pregnane-X receptor* (PXR), muscarine receptors and *sphingosine 1-phosphate receptor 2* (S1PR2) [70,71]. The activation of FXR stimulates the production of insulin and lowers the plasma concentration of cholesterol, triglycerides, and FFAs. It has been shown that the microbiota plays an essential role in high-fat-diet-induced obesity and hepatic steatosis and that this is mediated through FXR. When fed on a high-fat diet, no weight gain is observed in germfree mice. However, in mice deprived of FXR, the gut microbiota can contribute only to the increased glucose levels and impaired oral glucose tolerance, but does not affect weight gain [72].

TGR5 activation leads to greater energy consumption in brown adipose tissue, which can stop the development of IR and obesity. TRG5 takes part in the regulation of glucose and lipid metabolism and influences the immunological system [73,74]. The activation of TGR5 in endocrine cells stimulates the secretion of *glucagon-like peptide-1* (GLP-1), which in turn stimulates the secretion of insulin and lowers one’s appetite, thus preventing further food intake. The activation of TGR5 in liver macrophages contributes to the anti-inflammatory effect by inhibiting the production of cytokines and *nuclear factor* κB (NF-κB) activity [70,74,75,76]. It has been shown that the production of specific secondary BAs, (tauro)lithocholic acid, which is produced by the gut microbiota upon FXR-regulated microbial activity, can stimulate TGR5 and the subsequent metabolic cascade, leading to metabolic improvement [77].

In contrast to this beneficial effect of a group of secondary BAs, secondary BAs can increase the intestinal permeability and may contribute to DNA damage and an increase in the production of interleukin 1β (Il-1β), thus leading to hepatocellular cancer progression [76].

The increased permeability of the intestinal barrier leads to oxidative stress and bacterial translocation, together with the penetration of the intestinal wall by bacterial endotoxins, which in turn stimulates the attraction of immunological cells to adipose tissue and the development of its chronic inflammatory process. The most important endotoxin is lipopolysaccharide, a Gram-negative bacteria cell wall component. LPS stimulates the production and release of many proinflammatory cytokines, such as *tumor necrosis factor α* (TNFα), Il-1β, Il-6 and Il-8, which activate the signaling pathways of membrane-bound toll-like receptors (TLRs) and cytosol NOD-like receptors (NLRs). NLRs bind to inflammasomes, as well as fragments of intestinal microorganisms: LPS, lipoproteins, peptidoglycans, bacterial polysaccharides, viral RNA and bacterial wall proteins. Inflammasomes react to cell injury by activating caspase 1 and stimulating the release of proinflammatory cytokines. The activity of proinflammatory cytokines is noticeably higher in patients with NAFLD and obesity. Proinflammatory factors lead to IR, a higher plasma concentration of FFAs, the decreased production of adiponectin, increased production of leptin, hyperfagia and hyperlipidemia [76,77,78,79,80,81].

After binding to TLRs, LPS also activates macrophages and hepatic stellate cells (HSCs). The activation of macrophages further enhances the inflammatory process and triggers HSCs transformation into myofibroblasts that produce smooth muscle actin and collagen fibers. LPS makes macrophages and HSCs more susceptible to leptin, which has an important influence on the faster progression to fibrosis [80,81].

Finally, another important mechanism by which microbiota can contribute to the development of NAFLD and its progression to fibrosis and cirrhosis is the production of alcohol. The serum level of alcohol is significantly elevated in NASH patients compared to obese or lean controls [82]. Interestingly, increased endogenous ethanol is observed even in children suffering from NAFLD [83]. Higher ethanol production by the microbiota is typically associated with elevated levels of *Enterobacteriaceae*. In a Chinese cohort of NAFLD patients, 60% of patients had elevated levels of high-ethanol-producing *Klebsiella pneumonia*. The transfer of the microbiota containing this bacterium from a NASH patient to germ-free mice induced NAFLD in the animals [84].

## 4. Gut Dysbiosis and NAFLD

Dysbiosis is a condition in which the gut microbiota has changed from that of a healthy individual and is impaired or imbalanced. This change is not clearly described for any medical condition, but when comparing groups of patients and controls, dysbiosis appears as an overgrowth or reduction of certain microorganisms [11]. The increased prevalence of small intestine bacterial overgrowth (SIBO) in NAFLD patients was the first connection noticed in studies linking NAFLD and dysbiosis [85]. It has been shown that dysbiosis in NAFLD is connected to different stages of the disease. This means that dysbiosis in NASH patients differs from that with HCC development or with simple steatosis [86]. According to some studies, there are specific bacteria with potential implications in the protection against NAFLD development and obesity. Obese individuals have a less diverse microbiota, a phenomenon known as a low gene count (LGC). Their microbiota is characterized by a higher ratio of Gram-negative bacteria to Gram-positive bacteria. Although the literature data often discuss the decrease in the number of *Bacteroidetes* on account of the rise in the number of *Firmicutes*, which was demonstrated in one of the earliest studies addressing microbiota in obesity [48], recent data have shown that the *Firmicutes* to *Bacteroidetes* ratio is not a reliable marker of obesity-related dysbiosis [50]. It has already been mentioned that the extent and level of dysbiosis depends on numerous factors, which was highlighted in one study where patients were divided into two groups depending on their BMI and FMT was performed [87]. As the results showed a significantly better result in lean patients after FMT, the question arises whether microbiota disruption is more significant in the pathogenesis of NAFLD in lean patients compared to obese patients. The hypothesis that there are considerable changes in lean patients, in the form of a reduced number of bacteria that could have a good effect on the metabolism of glucose and lipids as well as inflammation in the intestines, had already been suggested in studies that preceded the one mentioned above. Microbiota shifts have to be addressed at a lower taxonomic resolution, and what appears to be relevant is that the microbiota of obese individuals is characterized by an increased abundance of *Ruminococcus gnavus*, a bacterium responsible for the degradation of mucus that is linked to the enhanced permeability of the intestinal barrier, and by the decreased abundance of the mucin degrader *Akkermansia muciniphila*, which is associated with the production of propionate and the stimulated production of mucus [50,64,65]. A reduced abundance of the anti-inflammatory butyrate-producing bacterium *Faecalibacterium prausnitzii* is associated with increased intestinal permeability [64,65,66]. The major carbohydrate metabolism products by *A. muciniphila*–propionate and by *F. prausnitzii*–butyrate are, together with acetate, jointly termed “short chain fatty acids” (SCFAs). SCFAs are associated with numerous beneficial health effects and it is known that they can stimulate the production of peptide YY—a hormone that inhibits the passage of food through the intestinal system by slowing down its motoric and thus enhances the absorption of nutrients [64,65,66]. In the liver, SCFAs are converted to triglycerides [67,68]. SCFAs are particularly relevant for energy metabolism as both propionate and butyrate play an important role in the intestinal gluconeogenesis. While butyrate activates glycogenesis on the gene expression level, propionate serves also as a signal molecule in a gut–brain neural circuit involving the fatty acid receptor FFAR3. By this mechanism, SCFAs have beneficial effects on glucose and energy homeostasis [69].

Diabetic patients were observed to have less butyrate-producing bacteria (*Clostridiales* sp. *SS3/4*, *Eubacterium recitale*, *F. prausnitzii* and *Roseburia intestinalis*) and more opportunistic pathogens [73,74]. The increased abundance of *Escherichia coli* and *Bacteriodes vulgatus* was found to be associated with the risk of advanced fibrosis in patients with NAFLD [70].

A study performed by Boursier et al. also searched for the correlation between gut microbiota dysbiosis and NAFLD and showed that gut microbiota dysbiosis can be used as a predictor of NAFLD severity and progression. By using the data of 57 NAFLD patients and multivariate analysis, the authors concluded that the *Bacteroides* is independently associated with NASH and *Ruminococcus* with fibrosis [88].

In the last few years, numerous works have been carried out in which intestinal dysbiosis is the common basis for the emergence of inflammatory bowel disease (IBD) and NAFLD, as well as the state of chronic inflammation. However, despite the obtained results, it is still not possible to accurately define common risk factors and pathogenesis. The type of IBD, the therapy applied to the patient, possible surgical treatment, the activity of the disease and its duration and the diet of the patient with IBD all play a major role in the occurrence of liver steatosis in patients with IBD. During times of high IBD activity, increased intestinal permeability contributes to the development of NAFLD through an increased IBD likelihood of the translocation of bacteria and their metabolites. [89]. The resection of the small intestine is also associated with the development of liver steatosis because after the removal of the part of the intestine that is affected by extensive inflammatory changes, an increase in body weight and an increase in the total mass of adipose tissue occurred in mice [90]. Furthermore, in cases where it is necessary, patients receive parenteral nutrition, the known side effect of which is a fatty liver [91]. A recently published study found a decrease in the presence of *Subdoligranulum*, *Parabacteroides* and *Fusicatenibacter* in patients with IBD compared to patients who also suffer from NAFLD in addition to IBD. *Alistipes*, *Odoribacter*, *Sutterella* and *Lachnospira* were more prevalent in the comparison of patients with NAFLD and those with NAFLD and IBD [92].

As evident from the presentation of selected studies, there is still no consensus regarding dysbiosis in NAFLD. Many technical factors contribute to this, including the NAFLD diagnosis criteria, the method of microbiota analysis, age and the geographic origin of patients. Despite the considerable heterogeneity of the results, NAFLD is associated with microbiota dysbiosis and given that there are already identified metabolic pathways by which the microbiota contributes to the NAFLD pathology, it is certain that the modulation of the microbiota is one of the most promising targets in the search for novel therapeutic options for NAFLD and associated CLD.

## 5. Modulation of Gut Microbiota in NAFLD

Even though the gut microbiota is relatively stable in adults, it is still susceptible to change. It is considered that the microbiota composition can be altered both in a positive or negative way due to different environmental factors, such as exposure to toxic compounds, diet or antibiotic consumption. Hence, it is possible to promote changes in the microbiota composition to provide a healthier profile. Substances with probiotic/prebiotic actions and, more recently, fecal microbiota transplantation (FMT) are the current strategies that have shown a modulatory capacity over dysbiosis and gut–liver axis activation [93,94]. The results of the effect of microbiota modulation on CLD can be measured by various means, such as hepatic histology (steatosis, lobular inflammation, balloon degeneration and fibrosis) and biochemical parameters (liver enzyme levels). Although liver enzymes are commonly used to assess the improvement in NASH, they are poor predictors of NASH and its prognosis. The efficacy of probiotics as well as alternative probiotic approaches have been explored in these clinical studies [11,49,60,95,96] and their effect on NAFLD was recently reviewed in a meta-analysis [50]. It is considered that probiotics can improve hepatic inflammation, histology, and function by retarding and reversing dysbiosis, as measured by biochemical markers, as well as in liver biopsy samples. Numerous beneficial effects of multistrain probiotics have been proven in many pathological conditions. It is also worth mentioning here the potential in reducing the systemic inflammatory state and resetting the composition of the microbiota to a composition similar to that of healthy individuals [97,98]. They have positive effects on the liver function and the biochemical lipid profile, which can be quantified through the values of liver enzymes as well as cholesterol and triglycerides reduction. Although probiotics could be used either alone or in conjunction with other NAFLD-targeted therapies, its potential interactions with other agents must still be studied. This is the reason why experts still hesitate to recommend the clinical use of probiotics for gut microbiota modification in patients with NAFLD. While it is generally perceived that further studies are needed to elucidate the role of probiotics [11,49,60,82,95,96], a recent meta-analysis indicates that the integrated results of 25 randomized clinical trials showed that probiotics and prebiotics significantly contribute to BMI reduction (0.37 kg/m^2^, *p* < 0.001), a decrease in hepatic enzymes (ALT, 6.9 U/L, AST, 4.6 U/L and c-GT, 7.9 U/L, *p* < 0.001), a decrease in serum cholesterol (10.1 mg/dL, *p* < 0.001), LDL-c (4.5 mg/dL; *p* < 0.001) and TAG (10.1 mg/dL; *p* < 0.001), but not in inflammation (assessed by TNF-a and CRP). Similar effects of prebiotics and probiotics on the BMI and liver enzymes were observed in another meta-analysis, while the lipid profile was improved only by prebiotic therapy [99].

There is a scarcity of studies that report the effect of probiotics on NAFLD beyond biochemical markers. One study reported a significant reduction in the intrahepatic triglyceride content as measured by proton-magnetic resonance spectroscopy as a result of 6 months’ mixed probiotic (Lepicol) consumption. The first report of histological improvement and changes in the microbial composition in response to a prebiotic compound in NASH patients was provided by a study that evaluated the effect of oligofructose consumption for 24 weeks. The significant reduction in steatosis and the NAFLD activity score were found in the prebiotic-treated group vs. the placebo, despite the small sample size. In addition, prebiotic administration resulted in reduced *Clostridium* cluster XI and increased the relative adundance of *Bifidobacteriu* [100].

### Fecal Microbiota Transplantation

Fecal microbiota transplantation (FMT) is defined as the transplantation of gut microbiota from healthy donors to a diseased recipient to restore the diversity of their gut microbiome. The history of FMT is old (more than 1500 years) and has its origins in China, where it was used for patients who had diarrhea or food poisoning. On the other hand, the first report of FMT in modern medicine is from 1958, when an American doctor used fecal enemas for the treatment of patients who had fulminant pseudomembranous enterocolitis because of antibiotic use. The used treatment was successful and the rapid resolution of symptoms was reported [101].

In the last decade, FMT has been analyzed as a possible therapy in many diseases. Currently, FMT is approved treatment only for recurrent infection caused by the bacterium *Clostridium difficile* [102]. To date, there is a large difference in FMT methods among centers worldwide, mainly regarding donor selection, stool preparation as well as in terms of the method of administration of the microbiota suspension, even though three years ago a European consensus report on FMT was published [103]. Usually, fecal microbiota for transplantation is obtained from volunteer donors. A donor can be a person who is known to the recipient, or it can be somebody that donates their stool to a frozen donor bank. Donors are carefully screened [104,105,106] by blood and stool tests for certain diseases such as gastrointestinal infections (viral hepatitis, HIV, COVID-19, and Epstein–Barr), intestinal parasites, inflammatory bowel disease, autoimmune diseases, inherited metabolic disorders, family history of cancer, recent antibiotic treatment, or use of controlled substances. These tests establish their eligibility as donors of stool. Any of these conditions may be a reason for excluding that person as a stool donor since the collected stool must be free of any infections or diseases.

Besides screening a healthy donor, it is also important that the donor is suitable [107,108] and that they match with the recipient [109].

Recipients of FMT must fulfill certain conditions in person, e.g., if a person is taking antibiotic treatment, it must be completed a few days before the FMT procedure [104,105]. The recipient may also be asked to fast for a few hours before the procedure and sometimes they can receive antacids or laxatives because their intestines should be practically free of contaminated fecal material before FMT in order to have a healthy graft. According to certain studies, loperamide is given to recipients to ensure that transferred fecal material stays for at least 4 h in the bowel [110,111].

The route of administration of fecal microbiota can be through either the upper gastrointestinal (GI) tract via a nasogastric tube [112,113] or oral capsule [114,115] or through the lower GI tract via a colonoscopy or enema [116].

Upper GI routes:

A nasogastric tube—a thin, flexible tube that is inserted through the patient’s nose into their stomach. The transferred fecal microbiota is inserted into the tube with a syringe. The nasogastric tube is usually placed before the procedure and removed after it [112,113,117].

Upper endoscopy—a slim tube (endoscope) is passed though the patient’s mouth or nose to their stomach or small intestine [118]. A longer nasojejunal tube may be used to deliver the sample into the small bowel [119,120]. This method is preferable for patients who, for some reason, cannot be submitted to a colonoscopy.

Oral capsule—capsules containing freeze-dried, live fecal microbiota are ingested through the patient’s mouth. These capsules are designed to stay intact until they reach the patient’s colon [114,115,121,122,123]. The newest Foof and Drug Administration (FDA)-approved treatment is an oral capsule containing *Firmicutes*-selected types of fecal source bacteria spores (fecal microbiota spores, live-brpk, and SER-109).

Lower GI routes:

Colonoscopy—a colonoscope is passed into the patient’s colon through their rectum and fecal microbiota is delivered through it, usually to the beginning of the large intestine or to the end of the small intestine. Historically, a colonoscopy has been the preferred method of fecal microbiota delivery [117].

Rectal enema—a tube is inserted into the patient’s rectum and transferred microbiota is delivered through it [124,125].

Preparation of fecal material

The optimal procedure for the preparation of fecal material is yet to be determined. It has been discussed whether it is better to use fresh or frozen fecal material. However, there are several clinical trials that have shown that the efficacy of the frozen FMT is the same as that of the fresh FMT for the treatment of *Clostridium difficile* infections [126,127]. Fresh fecal material needs to be processed within 6 h of obtaining it from the donor. It can be stored for 6 h at room temperature until additional processing. The processing amount should be approximately 50 g (not less than 30 g) and it should be mixed with sterile normal sodium chloride (approximately 150 mL) using a blender. To avoid the obstruction of the endoscope’s channel, the prepared mixture should be filtered through a filter or gauze to eliminate large residue. In the end, the obtained filtrate is filled into the syringes (60 mL) and administered to the patient’s GI tract. It is important that the final prepared material is properly labeled and stored at −80 °C. On the day of application, the frozen material is put in a 37 °C water bath where it is thawed and is then mixed with normal saline to obtain the necessary volume. The application should be performed within 6 h after thawing [104,105,106].

According to the literature, FMT is a relatively safe procedure and can be performed even in immunocompromised patients [99], although data regarding the very long-term risks (≥5 years) are limited. Theoretically there is a possibility of potentially harmful bacteria being transplanted, which has a negative effect that is not apparent for years [103]. Therefore, further prospective studies following patients that have received FMT are needed.

Given that FMT is currently recommended only for *C. difficile*-associated diarrhea, MetS and its associated disorders are a possible future target for FMT [95].

In the pioneering study assessing FMT efficacy on MetS, the authors analyzed the effects of infusing either autologous gut microbiota or gut microbiota from healthy lean donors to 18 recipients with MetS on the recipients’ microbiota composition and glucose metabolism [103]. They reported that six weeks after the infusion of microbiota from lean donors there was an amelioration of recipients’ IR along with an increase in the levels of butyrate-producing intestinal microbiota [128]. Interestingly, five years ago, Alang et al. [129] reported the case of a woman successfully treated with FMT because of recurrent *C. difficile* infection, who developed new-onset obesity after receiving stool from a healthy but overweight donor [130]. This case report suggests that both the various healthy and the diseased phenotypes can be transferred by microbiota. Although human studies regarding FMT in MetS are limited, data with animal models provide evidence for the possible use of FMT in MetS and disorders that are related to it. Table 2 summarizes recent human studies on FMT and intestinal dysbiosis.

Di Luccia B et al. [131] analyzed rats that were fed either a standard or high-fructose diet. Fructose-fed rats were treated with either antibiotics or fecal samples from control rats by oral gavage. The authors found that the fructose-rich diet induced markers of MetS as well as markers of inflammation and oxidative stress. All these markers were significantly reduced when the rats were treated with an antibiotic or fecal sample, which led to the reduction in plasma LPS. This was consequently associated with a lower representation of the *Coprococcus* and *Ruminococcus* genera in the group that was treated. More recently, Sung et al. [132] found an improvement in glucose homeostasis and reduced fat mass in animal models after FMT.

Ng et al. carried out a trial with obese patients who suffered from type-2 diabetes. FMT was performed and the results showed that lean gut microbiota engraftment, especially in combination with lifestyle modification, led to decreased lipid values and an improvement in liver stiffness [133].

Like MetS, there are limited data regarding the potential of FMT in NAFLD, most of which originates from animal studies that suggest a beneficial effect of FMT in the context of fatty liver disease. For example, the aim of the study by Chiu et al. [134] was to investigate the gut microbiota composition that could have an impact on the NAFLD progression in mice transplanted with feces from human NASH. Germ-free mice were inoculated with feces that were either from NASH patients or from healthy people. Mice were then fed with a standard diet or high-fat diet. The mice that were transplanted with feces from NASH patients had increased liver steatosis as well as inflammatory cell infiltration in contrast to mice transplanted with feces from healthy controls [132]. Furthermore, Zhou et al. [135] published the first analysis of FMT in a diet-induced NASH model. They found that FMT was associated with the increase in the concentration of fecal butyrate and the improvement of the tight junction of the small intestine. Finally, this study revealed that FMT attenuated steatohepatitis in mice by the modulation of the gut microbiota.

An interesting study was conducted on patients divided into lean and obese patients diagnosed with NAFLD [87]. One group of patients received probiotics (a *Bifidobacterium* viable preparation and *Lactobacillus acidophilus* capsules) and another was the FMT group. The results showed significantly reduced hepatic fat attenuation evaluated by FibroScan after FMT. Other findings such as blood lipids, liver function, and fat attenuation results did not show statistical differences before and after FMT. This result confirms earlier findings that the absence of a decrease in blood lipid levels or liver enzyme values does not necessarily mean that there is no effect of therapy, in this case, the effect of FMT. Since microbiota dysbiosis was proven in NAFLD patients, the impaired abundance of the gut microbiota had been improved after FMT. Interestingly, FMT had a greater impact on the microbial composition in lean NAFLD than in obese NAFLD patients, and this is supported by the fact that there was a significant reduction in the accumulation of fat in the liver. However, we must highlight previous findings regarding the microbiota in obese patients who have a higher abundance of SCFA-producing bacteria, and this can be associated with weight gain through the procurement of additional calories [136].

In one published pilot study the authors tested whether FMT from a lean, healthy donor given to NAFLD patients with MetS would result in the improvement of several parameters, including IR and intestinal permeability 6 weeks after FMT and the hepatic proton density fat fraction (PDFF), evaluated by magnetic resonance elastography (MRE) six months after FMT [135]. The study involved 21 patients and FMT was performed during a duodenoscopy. Liver steatosis was diagnosed by ultrasound, while the fibrosis stage was diagnosed using a transient elastography (i.e., FibroScan) in seven patients, MRE in five patients and liver biopsy in nine patients. NAFLD patients received an allogenic (*n* = 15) or autologous (*n* = 6) FMT and were randomly assigned. The authors did not find significant changes in the HOMA-IR score as a measurement of IR or hepatic PDFF in NAFLD patients who received the allogenic or autologous FMT. On the other hand, allogenic FMT NAFLD patients with elevated small intestinal permeability at the baseline had a significant reduction 6 weeks after FMT. One more interesting observation in this study is that although the authors did not discern changes in specific taxa within the fecal microbiota with allogenic FMT, they observed a trend toward an increase in the fecal microbiota diversity in those NAFLD patients who had an improvement in their intestinal permeability. Although this study did not find an improvement in IR, which is possibly due to the use of the HOMA-IR score as a measurement of IR, their result regarding the improvement in small intestinal permeability because of allogenic FMT is encouraging. Furthermore, the authors did not find a positive effect of FMT on the MRE findings in the follow-up period. This could be explained by the fact that changes in the gut microbiota associated with allogenic FMT did not persist up to the test period of 6 months. Thus, the question is whether repeated FMT would be sufficient to prevent the reversion of the gut microbiota to the baseline. The resilience of the microbiota could be triggered by persistent treatment, as observed in the persistent use of probiotics, which was associated with the improvement of hepatic proton density fat fraction PDFF at 6 months in one study [137].

Patients who are diagnosed with metabolic dysfunction-associated steatotic liver disease (MASLD) have a steatotic liver and, in most cases, diabetes mellitus type 2, as well as metabolic syndrome [138]. The gut microbiota features in this group of patients have shown an increased intestinal permeability and as for the composition of the microbiota, increased numbers of *γ-Proteobacteria* and decreased numbers of *Bacteroidetes* have been proved [139]. A group of researchers demonstrated that the amelioration of the disease severity can be achieved after FMT. In this case, from a vegan subject. The positive effect was demonstrated through the reduction in the necro-inflammatory histological score and lower hepatic inflammatory gene expression [140].

Although more research is needed, the results of FMT in the context of MetS and NAFLD in animal models are encouraging and FMT seems to be a promising treatment option that can reverse the intestinal dysbiosis associated with NAFLD. However, there are a few questions regarding the use of FMT in NAFLD patients. Firstly, what criteria for donor selection should be used for this patient’s population? The second question concerns the processing of feces (anaerobic vs. aerobic) and the method of administration: by the upper gastrointestinal or by the lower gastrointestinal tract route? Thirdly, as reported in the human study by Craven et al. [137], it remains to be determined whether single or multiple fecal microbiota transfers are needed and, in the case of multiple transfers, what should the time interval between the procedures be and for how long should they be performed. We must keep in mind that if multiple FMT procedures are performed using a colonoscopy, given that a colonoscopy is an invasive procedure, such action would not be a patient-friendly method for multiple use. For sure, the further development of noninvasive techniques such as the encapsulation of the donor gut microbiota that would allow oral administration could be a very good alternative method to an invasive endoscopy procedure. Nevertheless, the gut microbiota–liver axis capitalization presumably provides targets as a new method of treatment in the future for this CLD and the application of FMT in clinical practice requires further investigation due to the lack of data into humans. Given the fact that we still do not have an effective treatment for NAFLD/NASH patients, and the global importance and consequence of NAFLD, we trust there is an urgent need for further studies into the efficacy and safety of FMT in the context of NAFLD.

**Table 2:** Summarizing relevant human studies related to FMT and microbiome dysbiosis.

Trial Identifier	Type of Study and Design	Major Findings
PRJNA782181 [87]	- Controlled randomized clinical trial - In non-FMT group, patients received oral probiotics - In the FMT group, patients received FMT with donor stool (heterologous) via colonoscopy, followed by three enemas over 3 days	FMT improved intestinal microbiota dysbiosis, ↓ accumulation of fat in liver attenuating fatty liver disease. Differences found in gut microbiota and clinical characteristics between lean and obese NAFLD patients were significant. FMT showed better impact on gut microbiota restoration in lean NAFLD than in obese NAFLD patients. Clinical efficacy of FMT was higher in lean NAFLD than in obese NAFLD patients.
2. NCT02496390 [137]	- Randomized clinical trial - NAFLD patients were randomized in a ratio of 3:1 to either an allogenic (*n* = 15) or an autologous (*n* = 6) FMT delivered using an endoscope to the distal duodenum	No significant changes in HOMA-IR or hepatic PDFF in patients who received the allogenic or autologous FMT. FMT carried out HOMA-IR or hepatic PDFF. FMT had the potential to reduce small intestinal permeability in patients with NAFLD.
3. NL4189 (NTR4339) [140]	- Controlled double-blind randomized proof-of-principle study - Patients with hepatic steatosis on ultrasound were randomized to two study arms: lean vegan donor (allogenic *n* = 10) or own (autologous *n* = 11) FMT	Allogenic FMT resulted in improved necro-inflammatory histology. Observed significant changes in expression of hepatic genes involved in inflammation and lipid metabolism as well as the change in intestinal microbial community structure. FMT is associated with changes in plasma metabolites as well as markers of steatohepatitis.
4. NCT 02206841, (IRB No. 26-2017-48) [141]	- The analysis of gut microbiome profiles of 171 Asians with biopsy-proven NAFLD and 31 non-NAFLD controls - Subjects were classified into three subgroups according to histological spectra of NAFLD or fibrosis severity and analysis was performed using 16S rRNA sequencing	Significant alterations in microbiome diversity were found in non-obese, but not in obese, subjects with regard to fibrosis severity. ↑ stool bile acids and propionate, particularly in non-obese subjects with significant fibrosis. Confirmed role of the microbiome in the liver fibrosis pathogenesis, especially in non-obese subjects.
5. NL4189-NTR4339 [142]	- NAFLD patients divided into groups submitted to FMTs from either allogenic (*n* = 10) or autologous (*n* = 11) donors - Hepatic DNA methylation profiles obtained from paired liver biopsies (performed before and after FMTs for all participants). - Multi-omics machine learning approach was applied to identify changes in the gut microbiome, peripheral blood metabolome and liver DNA methylome, and analyzed cross-omics correlations	Vegan allogenic donor FMT induced specific differential changes when compared with autologous FMT in gut microbiota profiles, plasma metabolites and hepatic DNA methylation profiles of hepatic DNA. FMT caused modifications in gut microbiota composition, which caused extensive changes in plasma and liver DNA methylation profiles in individuals with NAFLD. It suggests that FMTs could induce changes in metaorganismal pathways from the gut bacteria to the liver.
6. NCT02636647 [143]	- Open-label, randomized clinical trial - 5-month follow-up in outpatient men with cirrhosis with recurrent HE on standard of care (SOC) was conducted - FMT-randomized patients 1:1 received 5 days of broad-spectrum antibiotic pretreatment, then a single FMT enema from the same donor with the optimal microbiota deficient in HE	FMT with antibiotic pretreatment was well tolerated. Improved cognition improved in the FMT, but not the SOC group. FMT reverted to baseline MELD score, which transiently worsened after antibiotic treatment. After the antibiotic treatment: ↓ beneficial taxa and microbial diversity reduction occurred with Proteobacteria expansion. After FMT: ↑ diversity and beneficial taxa. FMT from a rationally selected donor in cirrhosis with recurrent HE: ↓ hospitalizations and dysbiosis. ↑ cognition.
7. NCT02636647 [120]	- Open-label clinical trial - Patients with SAH-ACLF divided into two arms: standard of care arm (SOC) and FMT (freshly prepared stool suspension from healthy family member stool donors through a naso-jejunal tube in a single session)	In the FMT arm, the survival rate was significantly better at 28 and 90 days (100% versus 60%, *p* = 0.01; 53.84% versus 25%, *p* = 0.02).
8. NCT04749030 [144]	- Double-blinded, randomized, placebo-controlled pilot trial - Participants—type-1 diabetes adult patients with moderate-to-severe gastrointestinal symptoms randomized (1:1) to encapsulated FMT or placebo	FMT: Safe - Improved clinical outcomes for patients with type-1 diabetes suffering from bowel symptoms. - FMT group of patients’ median Gastrointestinal Symptom Rating Scale—Irritable Bowel Syndrome score from 58 (IQR 54–65) to 35 (32–48), while in patients receiving placebo the score was reduced from 64 (55–70) to 56 (50–77) (*p* = 0.01). The Irritable Bowel Syndrome Impact Scale score ameliorated from 108 (101–123) to 140 (124–161) in patients who were administered FMT and 77 (53–129) to 92 (54–142) in placebo group (*p* = 0.02). The Patient Assessment of Gastrointestinal Symptom Severity Index lowered from a median of 42 (28–47) to 25 (14–31) after FMT and 47 (31–69) to 41 (36–64) after placebo (*p* = 0.03).
9. NCT03127696 [133]	- Randomized, double-blind, placebo-controlled trial - Obese T2DM patients randomized in three parallel groups: FMT + lifestyle intervention (LSI), FMT alone or sham transplantation + LSI every 4 weeks for up to week 12 - Six healthy lean individuals were donors of FMT solution	Repeated FMTs: ↑ the level and duration of microbiota engraftment in obese patients with T2DM. Merging LSI and FMT: ↑ beneficial changes in microbiota of recipients as well as refinement in lipid profile and liver stiffness.
10. ACTRN12613000236796 [145]	- Multicenter, randomized, double-blind clinical trial - UC patients randomized to either anaerobically prepared pooled donor FMT (*n* = 38) or autologous FMT (*n* = 35) via colonoscopy followed by 2 enemas over 7 days	Steroid-free remission of U achieved in 32% participants receiving pooled donor FMT compared with 9% receiving autologous FMT (difference, 23% [95% CI, 4–42%]. Treatment with anaerobically prepared donor FMT compared with autologous FMT resulted in a higher likelihood of remission at 8 weeks.
11. NCT03426683 [146]	- In this study, we administered capsulized FMT to 22 patients with active UC to assess the efficiency of capsulized FMT and determine the specific bacteria and metabolite factors associated with the response to clinical remission	
12. ACTRN 12619000611123 [122]	- Randomized, double-blind, placebo-controlled trial - UC patients after 2 weeks of amoxicillin, metronidazole and doxycycline randomized to either oral lyophilized FMT or placebo capsules for 8 weeks	At week 8, in FMT group, 53% of patients were in corticosteroid-free clinical remission with endoscopic remission or response, as were 15% of patients in the placebo group (difference 38.3%, 95% CI 8.6–68.0; *p* = 0.027; odds ratio 5.0, 95% CI 1.8–14.1). Antibiotics followed by orally administered FMT were associated with the induction of remission in patients with active ulcerative colitis.
13. NCT01790061, NCT02560727 [147]	- Two clinical trials for moderate-to-severe ulcerative colitis - Both studies were pooled for analysis on the safety and efficacy of fecal microbiota transplantation in patients with ulcerative colitis over a 1-year follow-up	Both the method of preparation of microbiota from stool using the automatic system and the delivery method of colonic transendoscopic enteral tubing were associated with a lower rate of fecal microbiota transplantation-related adverse events. In total, 74.3% (81/109) and 51.4% (56/109) of patients achieved clinical response at 1 month and 3 months after step-up fecal microbiota transplantation, respectively. Fecal microbiota transplantation should be a safe and promising therapy for ulcerative colitis. The improved fecal microbiota preparation and colonic transendoscopic enteral tubing might reduce the rate of adverse events in ulcerative colitis.
14. ChiCTR2000030080 [148]	- Randomized pilot study - UC patients were randomized to the FMT group and the control group	The Mayo score was significantly decreased compared with that of the control group (*n* = 10) when reassessed at week 4 (*p* = 0.001) and week 8 (*p* = 0.019) after FMT. Additionally, 90% of patients in the FMT group and 50% of patients in the control group met the primary endpoint at week 8. After FMT, stool microbiota composition analysis indicated improved gut microbiota diversity. FMT significantly reduced the relative abundance of *Escherichia* and increased the relative abundance of *Prevotella* at the genus level.
15. NCT02299973 [149]	- Placebo-controlled double-blind randomized clinical trial - Refractory IBS patients randomized to single-dose nasojejunal administration of donor stools or autologous stools	FMT relieved symptoms compared with placebo (autologous transplant), although the effects decreased over 1 year. - Second FMT restored the response patients with a prior response. The response was associated with composition of the fecal microbiomes before FMT.
16. NCT02154867 [150]	- Placebo-controlled, double-blind, randomized clinical trial - Patients with moderate-to-severe IBS were randomized to active or placebo FMT, which was administered by colonoscopy	FMT induced significant symptom relief in patients with IBS.
17. NCT02788071 [151]	- Placebo-controlled, double-blind, randomized clinical trial - Adult patients with moderate-to-severe IBS were randomized to treatment with FMT capsules or placebo capsules	FMT group showed statistically significant improvement in stool frequency from during treatment to post-treatment and 1 month follow-up. There were no statistically significant differences observed for abdominal pain, stool frequency and stool form.
18. NCT03822299 [108]	- Placebo-controlled, double-blind, randomized clinical trial - Patients with IBS were randomized to placebo (own feces), 30 g FMT or 60 g FMT administered with gastroscope - FMT was obtained from one healthy well characterized donor	Reduction in the IBS symptoms 3 months after FMT (response) was observed in 23.6%, 76.9% (*p* < 0.0001) and 89.1% (*p* < 0.00001) of the patients who received placebo, 30 g FMT and 60 g FMT, respectively. In FMT group, significant improvements in fatigue and the quality of life were observed as well as changes in the intestinal bacterial profiles.
19. NTR1776 [130]	- Randomized clinical trial - Participants with metabolic syndrome were randomized to small intestinal infusion of allogenic or autologous microbiota from lean donors	Insulin sensitivity of recipients increased (median rate of glucose disappearance changed from 26.2 to 45.3 μmol/kg/min; *p* < 0.05) along with levels of butyrate-producing intestinal microbiota in period of 6 weeks after infusion of microbiota from lean donors.
20. NCT03477916 [152]	- Placebo-controlled, double-blind, randomized clinical trial - Tested the application of daily fiber supplementation as an adjunct to FMT therapy to modulate cardiometabolic outcomes - Patients with severe obesity and metabolic syndrome were randomized trial to receiving oral FMT-high-fermentable (HF) and FMT-low-fermentable (LF) fiber supplements	After 6 weeks: - Only patients in the FMT-LF group had significant improvements in homeostatic model assessment (HOMA2-IR). - Patients in FMT-HF, HF or LF groups showed no significant improvement in HOMA2-IR.
21. ACTRN12615001351505 [153]	- Placebo-controlled, randomized, double-masked clinical trial - Adolescents aged 14–18 with body mass index of ≥30 were randomized to FMT oral-encapsulated fecal microbiome from 4 healthy lean donors or to saline placebo	- Reductions in android-to-gynoid fat ratio in the FMT vs. placebo group were observed at 6, 12 and 26 weeks. - Profiling of gut microbiome showed a shift in community composition among the FMT group, maintained up to 12 weeks. - Post hoc exploratory analyses among participants with metabolic syndrome at baseline showed that FMT led to greater resolution of this condition (18 to 4) compared with placebo (13 to 10) by 26 weeks. - No effects were observed on insulin sensitivity, liver function, lipid profile, inflammatory markers, blood pressure, total body fat percentage, gut health and health-related quality of life.

FMT—Fecal Microbiota Transplantation.

The number of studies conducted on humans is still not large enough to be able to draw relevant results that could become part of the guidelines in the treatment of NAFLD. In addition, whether there is a difference in the way FMT is implemented in relation to the results and the eventual benefit achieved in the patient needs to be examined. Despite many studies that we have included in this article, the mechanism of how dysbiosis is related to the development of NAFLD cannot be fully clarified and, therefore, we believe that more research is needed that should be directed towards laying the foundations for a personalized approach to the patient.

## 6. Conclusions

Based on the results of profiling the composition of the microbiota of patients with diseases and conditions that are part of the metabolic syndrome, as well as NAFLD itself, we can conclude that there are specific differences in the composition of the microbiota. The result is a reduced production of favorable metabolites involved in the maintenance of metabolic homeostasis. Since the microbiota makes a significant contribution in the pathogenesis of NAFLD and MAFLD, it represents one of possible therapeutic targets. Changes in the microbial composition can be mitigated by transplanting fecal microbiota, but the results of the preclinical research need to be applied to humans in order to know which bacterial species and strains should be specifically affected in order to obtain the desired results in the form of alleviating disease symptoms and preventing their progression. In the case of NAFLD, this is particularly important due to the limited choice of available therapy, all for the purpose of preventing the development of liver fibrosis and, consequently, the possible development of hepatocellular liver cancer.

## References

[1] Chen H.T., Huang H.L., Li Y.Q., Xu H.M., Zhou Y.J. (2020). Therapeutic advances in non-alcoholic fatty liver disease: A microbiota-centered view. World J. Gastroenterol..

[2] Tang A., Ng C.H., Phang P.H., Chan K.E., Chin Y.H., Fu C.E., Zeng R.W., Xiao J., Tan D.J.H., Quek J. (2023). Comparative Burden of Metabolic Dysfunction in Lean NAFLD vs Non-lean NAFLD—A Systematic Review and Meta-analysis. Clin. Gastroenterol. Hepatol..

[3] Younossi Z.M., Golabi P., Paik J.M., Henry A., Van Dongen C., Henry L. (2023). The global epidemiology of nonalcoholic fatty liver disease (NAFLD) and nonalcoholic steatohepatitis (NASH): A systematic review. Hepatology.

[4] Teng M.L., Ng C.H., Huang D.Q., Chan K.E., Tan D.J., Lim W.H., Yang J.D., Tan E., Muthiah M.D. (2023). Global incidence and prevalence of nonalcoholic fatty liver disease. Clin. Mol. Hepatol..

[5] Lechner S., Yee M., Limketkai B.N., Pham E.A. (2020). Fecal Microbiota Transplantation for Chronic Liver Diseases: Current Understanding and Future Direction. Dig. Dis. Sci..

[6] Cortez-Pinto H., Camilo M.E., Baptista A., De Oliveira A.G., De Moura M.C. (1999). Non-alcoholic fatty liver: Another feature of the metabolic syndrome?. Clin. Nutr..

[7] Eslam M., Sanyal A.J., George J. (2020). International Consensus Panel. MAFLD: A Consensus-Driven Proposed Nomenclature for Metabolic Associated Fatty Liver Disease. Gastroenterology.

[8] Eslam M., Newsome P.N., Sarin S.K., Anstee Q.M., Targher G., Romero-Gomez M., Zelber-Sagi S., Wai-Sun Wong V., Dufour J.F., Schattenberg J.M. (2020). A new definition for metabolic dysfunction-associated fatty liver disease: An international expert consensus statement. J. Hepatol..

[9] Boccatonda A., Andreetto L., D’Ardes D., Cocco G., Rossi I., Vicari S., Schiavone C., Cipollone F., Guagnano M.T. (2023). From NAFLD to MAFLD: Definition, Pathophysiological Basis and Cardiovascular Implications. Biomedicines.

[10] Miele L., Marrone G., Lauritano C., Cefalo C., Gasbarrini A., Day C., Grieco A. (2013). Gut-liver Axis and Microbiota in NAFLD: Insight Pathophysiology for Novel Therapeutic Target. Curr. Pharm. Des..

[11] Perumpail B.J., Li A.A., John N., Sallam S., Shah N.D., Kwong W., Cholankeril G., Kim D., Ahmed A. (2019). The Therapeutic Implications of the Gut Microbiome and Probiotics in Patients with NAFLD. Diseases.

[12] Hou K., Wu Z.X., Chen X.Y., Wang J.Q., Zhang D., Xiao C., Zhu D., Koya J.B., Wei L., Li J. (2022). Microbiota in health and diseases. Signal Transduct. Target. Ther..

[13] Arab J.P., Martin-Mateos R.M., Shah V.H. (2018). Gut–liver axis, cirrhosis and portal hypertension: The chicken and the egg. Hepatol. Int..

[14] Hrncir T. (2022). Gut Microbiota Dysbiosis: Triggers, Consequences, Diagnostic and Therapeutic Options. Microorganisms.

[15] Hu H., Lin A., Kong M., Yao X., Yin M., Xia H., Ma J., Liu H. (2020). Intestinal microbiome and NAFLD: Molecular insights and therapeutic perspectives. J. Gastroenterol..

[16] Henao-Mejia J., Elinav E., Jin C., Hao L., Mehal W.Z., Strowig T., Thaiss C.A., Kau A.L., Eisenbarth S.C., Jurczak M.J. (2012). Inflammasome-mediated dysbiosis regulates progression of NAFLD and obesity. Nature.

[17] Wang L., Cao Z.M., Zhang L.L., Li J.M., Lv W.L. (2022). The Role of Gut Microbiota in Some Liver Diseases: From an Immunological Perspective. Front. Immunol..

[18] Duarte-Mata D.I., Salinas-Carmona M.C. (2023). Antimicrobial peptides’ immune modulation role in intracellular bacterial infection. Front. Immunol..

[19] Taranto M.P., Perez-Martinez G., Font de Valdez G. (2006). Effect of bile acid on the cell membrane functionality of lactic acid bacteria for oral administration. Res. Microbiol..

[20] Ploton M., Mazuy C., Gheeraert C., Dubois V., Berthier A., Dubois-Chevalier J., Maréchal X., Bantubungi K., Diemer H., Cianférani S. (2018). The nuclear bile acid receptor FXR is a PKA- and FOXA2-sensitive activator of fasting hepatic gluconeogenesis. J. Hepatol..

[21] Adorini L., Trauner M. (2023). FXR agonists in NASH treatment. J. Hepatol..

[22] Anand S., Mande S.S. (2022). Host-microbiome interactions: Gut-Liver axis and its connection with other organs. npj Biofilms Microbiomes.

[23] Larabi A.B., Masson H.L.P., Bäumler A.J. (2023). Bile acids as modulators of gut microbiota composition and function. Gut Microbes.

[24] Wang N., Huo Y., Gao X., Li Y., Cheng F., Zhang Z. (2024). Lead exposure exacerbates liver injury in high-fat diet-fed mice by disrupting the gut microbiota and related metabolites. Food Funct..

[25] Lang S., Schnabl B. (2020). Microbiota and Fatty Liver Disease—The Known, the Unknown, and the Future. Cell Host Microbe.

[26] Rochlani Y., Pothineni N.V., Kovelamudi S., Mehta J.L. (2017). Metabolic syndrome: Pathophysiology, management, and modulation by natural compounds. Ther. Adv. Cardiovasc. Dis..

[27] Griffin M.E., Marcucci M.J., Cline G.W., Bell K., Barucci N., Lee D., Goodyear L.J., Kraegen E.W., White M.F., Shulman G.I. (2000). Free fatty acid-induced insulin resistance is associated with activation of protein kinase C θ and alterations in the insulin signaling cascade. Diabetes.

[28] Tripathy D., Mohanty P., Dhindsa S., Syed T., Ghanim H., Aliada A., Dandona P. (2003). Elevation of Free Fatty Acids Induces Inflammation and Impairs Vascular Reactivity in Healthy Subjects. Diabetes.

[29] Soták M., Clark M., Suur B.E., Börgeson E. (2025). Inflammation and resolution in obesity. Nat. Rev. Endocrinol..

[30] Masenga S.K., Kabwe L.S., Chakulya M., Kirabo A. (2023). Mechanisms of Oxidative Stress in Metabolic Syndrome. Int. J. Mol. Sci..

[31] Rosendo-Silva D., Viana S., Carvalho E., Reis F., Matafome P. (2023). Are gut dysbiosis, barrier disruption, and endotoxemia related to adipose tissue dysfunction in metabolic disorders? Overview of the mechanisms involved. Intern. Emerg. Med..

[32] Matar A., Damianos J.A., Jencks K.J., Camilleri M. (2024). Intestinal Barrier Impairment, Preservation, and Repair: An Update. Nutrients.

[33] Tilg H., Adolph T.E., Trauner M. (2022). Gut-liver axis: Pathophysiological concepts and clinical implications. Cell Metab..

[34] Cani P.D., Amar J., Iglesias M.A., Poggi M., Knauf C., Bastelica D., Neyrinck A.M., Fava F., Tuohy K.M., Chabo C. (2007). Metabolic endotoxemia initiates obesity and insulin resistance. Diabetes.

[35] Al Bander Z., Nitert M.D., Mousa A., Naderpoor N. (2020). The Gut Microbiota and Inflammation: An Overview. Int. J. Environ. Res. Public Health.

[36] Tomas J., Mulet C., Saffarian A., Cavin J.-B., Ducroc R., Regnault B., Kun Tan C., Duszka K., Burcelin R., Wahli W. (2016). High-fat diet modifies the PPAR-γ pathway leading to disruption of microbial and physiological ecosystem in murine small intestine. Proc. Natl. Acad. Sci. USA.

[37] Alhabeeb H., Alfaiz A., Kutbi E., Alshahrani D., Alsuhail A., Alrajhi S., Alotaibi N., Alotaibi K., AlAmri S., Alghamdi S. (2021). Gut hormones in health and obesity: The upcoming role of short chain fatty acids. Nutrients.

[38] Dalby M.J., Aviello G., Ross A.W., Walker A.W., Barrett P., Morgan P.J. (2018). Diet induced obesity is independent of metabolic endotoxemia and TLR4 signalling, but markedly increases hypothalamic expression of the acute phase protein, SerpinA3N. Sci. Rep..

[39] Crudele L., Gadaleta R.M., Cariello M., Moschetta A. (2023). Gut microbiota in the pathogenesis and therapeutic approaches of diabetes. eBioMedicine.

[40] Karlsson F.H., Tremaroli V., Nookaew I., Bergström G., Behre C.J., Fagerberg B., Nielsen J., Bäckhed F. (2013). Gut metagenome in European women with normal, impaired and diabetic glucose control. Nature.

[41] Koh A., Molinaro A., Ståhlman M., Khan M.T., Schmidt C., Mannerås-Holm L., Wu H., Carreras A., Jeong H., Olofsson L.E. (2018). Microbially Produced Imidazole Propionate Impairs Insulin Signaling through mTORC1. Cell.

[42] de Groot P.F., Frissen M.N., de Clercq N.C., Nieuwdorp M. (2017). Fecal microbiota transplantation in metabolic syndrome: History, present and future. Gut Microbes.

[43] Koren O., Spor A., Felin J., Fåk F., Stombaugh J., Tremaroli V., Behre C.J., Knight R., Fagerberg B., Ley R.E. (2011). Human oral, gut, and plaque microbiota in patients with atherosclerosis. Proc. Natl. Acad. Sci. USA.

[44] Cindoruk M., Cirak M.Y., Unal S., Karakan T., Erkan G., Engin D., Dumlu S., Turet S. (2008). Identification of Helicobacter species by 16S rDNA PCR and sequence analysis in human liver samples from patients with various etiologies of benign liver diseases. Eur. J. Gastroenterol. Hepatol..

[45] Zulian A., Cancello R., Ruocco C., Gentilini D., Di Blasio A.M., Danelli P., Cesana E., Invitti C. (2013). Differences in Visceral Fat and Fat Bacterial Colonization between Ulcerative Colitis and Crohn’s Disease. An In Vivo and In Vitro Study. PLoS ONE.

[46] Pedersen H.K., Gudmundsdottir V., Nielsen H.B., Hyotylainen T., Nielsen T., Jensen B.A.H., Forslund K., Hildebrand F., Prifti E., Falony G. (2016). Human gut microbes impact host serum metabolome and insulin sensitivity. Nature.

[47] Shin N.R., Lee J.C., Lee H.Y., Kim M.S., Whon T.W., Lee M.S., Bae J.W. (2014). An increase in the Akkermansia spp. population induced by metformin treatment improves glucose homeostasis in diet-induced obese mice. Gut.

[48] Galic S., Oakhill J.S., Steinberg G.R. (2010). Adipose tissue as an endocrine organ. Mol. Cell. Endocrinol..

[49] Li J., Lin S., Vanhoutte P.M., Woo C.W., Xu A. (2016). Akkermansia muciniphila protects against atherosclerosis by preventing metabolic endotoxemia-induced inflammation in Apoe-/- Mice. Circulation.

[50] Depommier C., Everard A., Druart C., Plovier H., Van Hul M., Vieira-Silva S., Falony G., Raes J., Maiter D., Delzenne N.M. (2019). Supplementation with Akkermansia muciniphila in overweight and obese human volunteers: A proof-of-concept exploratory study. Nat. Med..

[51] Turnbaugh P.J., Ley R.E., Mahowald M.A., Magrini V., Mardis E.R., Gordon J.I. (2006). An obesity-associated gut microbiome with increased capacity for energy harvest. Nature.

[52] Liu B.N., Liu X.T., Liang Z.H., Wang J.H. (2021). Gut microbiota in obesity. World J. Gastroenterol..

[53] Schellekens H., Torres-Fuentes C., van de Wouw M., Long-Smith C.M., Mitchell A., Strain C., Berding K., Bastiaanssen T.F.S., Rea K., Golubeva A.V. (2021). Bifidobacterium longum counters the effects of obesity: Partial successful translation from rodent to human. eBioMedicine.

[54] Crovesy L., Ostrowski M., Ferreira D.M.T.P., Rosado E.L., Soares-Mota M. (2017). Effect of Lactobacillus on body weight and body fat in overweight subjects: A systematic review of randomized controlled clinical trials. Int. J. Obes..

[55] Salamon D., Sroka-Oleksiak A., Kapusta P., Szopa M., Mrozińska S., Ludwig-Słomczyńska A.H., Wołkow P.P., Bulanda M., Klupa T., Małecki M.T. (2018). Characteristics of the gut microbiota in adult patients with type 1 and 2 diabetes based on the analysis of a fragment of 16S rRNA gene using next-generation sequencing. Pol. Arch. Intern. Med..

[56] Yamaguchi Y., Adachi K., Sugiyama T., Shimozato A., Ebi M., Ogasawara N., Funaki Y., Goto C., Sasaki M., Kasugai K. (2016). Association of Intestinal Microbiota with Metabolic Markers and Dietary Habits in Patients with Type 2 Diabetes. Digestion.

[57] Sedighi M., Razavi S., Navab-Moghadam F., Khamseh M.E., Alaei-Shahmiri F., Mehrtash A., Amirmozafari N. (2017). Comparison of gut microbiota in adult patients with type 2 diabetes and healthy individuals. Microb. Pathog..

[58] Gao R., Zhu C., Li H., Yin M., Pan C., Huang L., Kong C., Wang X., Zhang Y., Qu S. (2018). Dysbiosis Signatures of Gut Microbiota Along the Sequence from Healthy, Young Patients to Those with Overweight and Obesity. Obesity.

[59] Lippert K., Kedenko L., Antonielli L., Kedenko I., Gemeier C., Leitner M., Kautzky-Willer A., Paulweber B., Hackl E. (2017). Gut microbiota dysbiosis associated with glucose metabolism disorders and the metabolic syndrome in older adults. Benef. Microbes.

[60] Plovier H., Everard A., Druart C., Depommier C., Van Hul M., Geurts L., Chilloux J., Ottman N., Duparc T., Lichtenstein L. (2017). A purified membrane protein from Akkermansia muciniphila or the pasteurized bacterium improves metabolism in obese and diabetic mice. Nat. Med..

[61] Moreno-Indias I., Sánchez-Alcoholado L., Pérez-Martínez P., Andrés-Lacueva C., Cardona F., Tinahones F., Queipo-Ortuño M.I. (2016). Red wine polyphenols modulate fecal microbiota and reduce markers of the metabolic syndrome in obese patients. Food Funct..

[62] Gargari G., Deon V., Taverniti V., Gardana C., Denina M., Riso P., Guardamagna O., Guglielmetti S. (2018). Evidence of dysbiosis in the intestinal microbial ecosystem of children and adolescents with primary hyperlipidemia and the potential role of regular hazelnut intake. FEMS Microbiol. Ecol..

[63] Park J.H., Kotani T., Konno T., Setiawan J., Kitamura Y., Imada S., Usui Y., Hatano N., Shinohara M., Saito Y. (2016). Promotion of intestinal epithelial cell turnover by commensal bacteria: Role of short-chain fatty acids. PLoS ONE.

[64] Magne F., Gotteland M., Gauthier L., Zazueta A., Pesoa S., Navarrete P., Balamurugan R. (2020). The firmicutes/bacteroidetes ratio: A relevant marker of gut dysbiosis in obese patients?. Nutrients.

[65] Donnelly K.L., Smith C.I., Schwarzenberg S.J., Jessurun J., Boldt M.D., Parks E.J. (2005). Sources of fatty acids stored in liver and secreted via lipoproteins in patients with nonalcoholic fatty liver disease. J. Clin. Investig..

[66] He X., Ji G., Jia W., Li H. (2016). Gut microbiota and nonalcoholic fatty liver disease: Insights on mechanism and application of metabolomics. Int. J. Mol. Sci..

[67] Guo X., Li S., Zhang J., Wu F., Li X., Wu D., Zhang M., Ou Z., Jie Z., Yan Q. (2017). Genome sequencing of 39 Akkermansia muciniphila isolates reveals its population structure, genomic and functional diverisity, and global distribution in mammalian gut microbiotas. BMC Genom..

[68] Raj A., Shanahan E.R., Fletcher L.M., Tran C., Bhat P., Morrison M., Holtmann G., Macdonald G. (2016). Increased small intestinal permeability in chronic liver disease is associated with reduced abundance of Faecalibacterium prausnitzii in the terminal ileum Mucosa. Hepatology.

[69] De Vadder F., Kovatcheva-Datchary P., Goncalves D., Vinera J., Zitoun C., Duchampt A., Bäckhed F., Mithieux G. (2014). Microbiota-generated metabolites promote metabolic benefits via gut-brain neural circuits. Cell.

[70] Erejuwa O.O., Sulaiman S.A., Ab Wahab M.S. (2014). Modulation of gut microbiota in the management of metabolic disorders: The prospects and challenges. Int. J. Mol. Sci..

[71] Wang J., Qin J., Li Y., Cai Z., Li S., Zhu J., Liang S., Zhang W., Guan Y., Shen D. (2012). A metagenome-wide association study of gut microbiota in type 2 diabetes. Nature.

[72] Parséus A., Sommer N., Sommer F., Caesar R., Molinaro A., Stahlman M., Greiner T.U., Perkins R., Bäckhed F. (2017). Microbiota-induced obesity requires farnesoid X receptor. Gut.

[73] Le Chatelier E., Nielsen T., Qin J., Prifti E., Hildebrand F., Falony G., Almeida M., Arumugam M., Batto J.M., Kennedy S. (2013). Richness of human gut microbiome correlates with metabolic markers. Nature.

[74] Zhang C., Zhang M., Wang S., Han R., Cao Y., Hua W., Mao Y., Zhang X., Pang X., Wei C. (2010). Interactions between gut microbiota, host genetics and diet relevant to development of metabolic syndromes in mice. ISME J..

[75] Tilg H., Moschen A.R. (2014). Microbiota and diabetes: An evolving relationship. Gut.

[76] Loomba R., Seguritan V., Li W., Long T., Klitgord N., Bhatt A., Dulai P.S., Caussy C., Bettencourt R., Highlander S.K. (2017). Gut Microbiome-Based Metagenomic Signature for Non-invasive Detection of Advanced Fibrosis in Human Nonalcoholic Fatty Liver Disease. Cell Metab..

[77] Pathak P., Xie C., Nichols R.G., Ferrell J.M., Boehme S., Krausz K.W., Patterson A.D., Gonzalez F.J., Chiang J.Y.L. (2018). Intestine farnesoid X receptor agonist and the gut microbiota activate G-protein bile acid receptor-1 signaling to improve metabolism. Hepatology.

[78] Yu S.J., Kim W., Kim D., Yoon J.H., Lee K., Kim J.H., Cho E.J., Lee J.H., Kim H.Y., Kim Y.J. (2015). Visceral obesity predicts significant fibrosis in patients with nonalcoholic fatty liver disease. Medicine.

[79] Arab J.P., Karpen S.J., Dawson P.A., Arrese M., Trauner M. (2017). Bile acids and nonalcoholic fatty liver disease: Molecular insights and therapeutic perspectives. Hepatology.

[80] Guo C., Xie S., Chi Z., Zhang J., Liu Y., Zhang L., Zheng M., Zhang X., Xia D., Ke Y. (2016). Bile Acids Control Inflammation and Metabolic Disorder through Inhibition of NLRP3 Inflammasome. Immunity.

[81] Bourzac K. (2014). Microbiome: The bacterial tightrope. Nature.

[82] Zhu L., Baker S.S., Gill C., Liu W., Alkhouri R., Baker R.D., Gill S.R. (2013). Characterization of gut microbiomes in nonalcoholic steatohepatitis (NASH) patients: A connection between endogenous alcohol and NASH. Hepatology.

[83] Michail S., Lin M., Frey M.R., Fanter R., Paliy O., Hilbush B., Reo N.V. (2015). Altered gut microbial energy and metabolism in children with non-alcoholic fatty liver disease. FEMS Microbiol. Ecol..

[84] Yuan J., Chen C., Cui J., Lu J., Yan C., Wei X., Zhao X., Li N., Li S., Xue G. (2019). Fatty Liver Disease Caused by High-Alcohol-Producing Klebsiella pneumoniae. Cell Metab..

[85] Schuppan D., Surabattula R., Wang X.Y. (2018). Determinants of fibrosis progression and regression in NASH. J. Hepatol..

[86] Buzzetti E., Pinzani M., Tsochatzis E.A. (2016). The multiple-hit pathogenesis of non-alcoholic fatty liver disease (NAFLD). Metabolism.

[87] Xue L., Deng Z., Luo W., He X., Chen Y. (2022). Effect of Fecal Microbiota Transplantation on Non-Alcoholic Fatty Liver Disease: A Randomized Clinical Trial. Front. Cell. Infect. Microbiol..

[88] Boursier J., Mueller O., Barret M., Machado M., Fizanne L., Araujo-Perez F., Guy C.D., Seed P.C., Rawls J.F., David L.A. (2016). The severity of nonalcoholic fatty liver disease is associated with gut dysbiosis and shift in the metabolic function of the gut microbiota. Hepatology.

[89] Bessissow T., Le N.H., Rollet K., Afif W., Bitton A., Sebastiani G. (2016). Incidence and Predictors of Nonalcoholic Fatty Liver Disease by Serum Biomarkers in Patients with Inflammatory Bowel Disease. Inflamm. Bowel Dis..

[90] Hoffmann P., Jung V., Gauss A., Behnisch R. (2020). Prevalence and risk factors of nonalcoholic fatty liver disease in patients with inflammatory bowel diseases: A cross-sectional and longitudinal analysis. World J. Gastroenterol..

[91] Mihajlovic M., Rosseel Z., De Waele E., Vinken M. (2024). Parenteral nutrition-associated liver injury: Clinical relevance and mechanistic insights. Toxicol. Sci..

[92] De Caro C., Spagnuolo R., Quirino A., Mazza E., Carrabetta F., Maurotti S., Cosco C., Bennardo F., Roberti R., Russo E. (2024). Gut Microbiota Profile Changes in Patients with Inflammatory Bowel Disease and Non-Alcoholic Fatty Liver Disease: A Metagenomic Study. Int. J. Mol. Sci..

[93] Yoo S., Jung S.C., Kwak K., Kim J.S. (2024). The Role of Prebiotics in Modulating Gut Microbiota: Implications for Human Health. Int. J. Mol. Sci..

[94] Nezhadi J., Fadaee M., Ahmadi S., Kafil H.S. (2024). Microbiota transplantation. Heliyon.

[95] Porras D., Nistal E., Martínez-Flórez S., González-Gallego J., García-Mediavilla M.V., Sánchez-Campos S. (2018). Intestinal microbiota modulation in obesity-related non-alcoholic fatty liver disease. Front. Physiol..

[96] Mouzaki M., Comelli E.M., Arendt B.M., Bonengel J., Fung S.K., Fischer S.E., McGilvray I.D., Allard J.P. (2013). Intestinal microbiota in patients with nonalcoholic fatty liver disease. Hepatology.

[97] Kobyliak N., Abenavoli L., Mykhalchyshyn G., Kononenko L., Boccuto L., Kyriienko D., Dynnyk O. (2018). A multi-strain probiotic reduces the fatty liver index, cytokines and aminotransferase levels in NAFLD patients: Evidence from a randomized clinical trial. J. Gastrointest. Liver Dis..

[98] Derosa G., Guasti L., D’Angelo A., Martinotti C., Valentino M.C., Di Matteo S., Bruno G.M., Maresca A.M., Gaudio G.V., Maffioli P. (2022). Probiotic Therapy with VSL#3^®^ in Patients with, NAFLD: A Randomized Clinical Trial. Front. Nutr..

[99] Wong V.W.S., Wong G.L.H., Chim A.M.L., Chu W.C.W., Yeung D.K.W., Li K.C.T., Chan H.L. (2013). Treatment of nonalcoholic steatohepatitis with probiotics. A proof-of-concept study. Ann. Hepatol..

[100] Bomhof M.R., Parnell J.A., Ramay H.R., Crotty P., Rioux K.P., Probert C.S., Jayakumar S., Raman M., Reimer R.A. (2019). Histological improvement of non-alcoholic steatohepatitis with a prebiotic: A pilot clinical trial. Eur. J. Nutr..

[101] Surawicz C.M., Brandt L.J., Binion D.G., Ananthakrishnan A.N., Curry S.R., Gilligan P.H., McFarland L.V., Mellow M., Zuckerbraun B.S. (2013). Guidelines for diagnosis, treatment, and prevention of clostridium difficile infections. Am. J. Gastroenterol..

[102] Ebrahimzadeh Leylabadlo H., Ghotaslou R., Samadi Kafil H., Feizabadi M.M., Moaddab S.Y., Farajnia S., Sheykhsaran E., Sanaie S., Shanehbandi D., Bannazadeh Baghi H. (2020). Non-alcoholic fatty liver diseases: From role of gut microbiota to microbial-based therapies. Eur. J. Clin. Microbiol. Infect. Dis..

[103] König J., Siebenhaar A., Högenauer C., Arkkila P., Nieuwdorp M., Norén T., Ponsioen C.Y., Rosien U., Rossen N.G., Satokari R. (2017). Consensus report: Faecal microbiota transfer—Clinical applications and procedures. Aliment. Pharmacol. Ther..

[104] Kassam Z., Dubois N., Ramakrishna B., Ling K., Qazi T., Smith M., Kelly C.R., Fischer M., Allegretti J.R., Budree S. (2019). Donor Screening for Fecal Microbiota Transplantation. N. Engl. J. Med..

[105] Cammarota G., Ianiro G., Tilg H., Rajilić-Stojanović M., Kump P., Satokari R., Sokol H., Arkkila P., Pintus C., Hart A. (2017). European consensus conference on faecal microbiota transplantation in clinical practice. Gut.

[106] Keller J.J., Ooijevaar R.E., Hvas C.L., Terveer E.M., Lieberknecht S.C., Högenauer C., Arkkila P., Sokol H., Gridnyev O., Mégraud F. (2021). A standardised model for stool banking for faecal microbiota transplantation: A consensus report from a multidisciplinary UEG working group. United Eur. Gastroenterol. J..

[107] Porcari S., Benech N., Valles-Colomer M., Segata N., Gasbarrini A., Cammarota G., Sokol H., Ianiro G. (2023). Key determinants of success in fecal microbiota transplantation: From microbiome to clinic. Cell Host Microbe.

[108] El-Salhy M., Hatlebakk J.G., Gilja O.H., Bråthen Kristoffersen A., Hausken T. (2020). Efficacy of faecal microbiota transplantation for patients with irritable bowel syndrome in a randomised, double-blind, placebo-controlled study. Gut.

[109] He R., Li P., Wang J., Cui B., Zhang F., Zhao F. (2022). The interplay of gut microbiota between donors and recipients determines the efficacy of fecal microbiota transplantation. Gut Microbes.

[110] Goldenberg S.D., Batra R., Beales I., Digby-Bell J.L., Irving P.M., Kellingray L., Narbad A., Franslem-Elumogo N. (2018). Comparison of Different Strategies for Providing Fecal Microbiota Transplantation to Treat Patients with Recurrent Clostridium difficile Infection in Two English Hospitals: A Review. Infect. Dis. Ther..

[111] Brandt L.J., Aroniadis O.C. (2013). An overview of fecal microbiota transplantation: Techniques, indications, and outcomes. Gastrointest. Endosc..

[112] Bakken J.S., Borody T., Brandt L.J., Brill J.V., Demarco D.C., Franzos M.A., Kelly C., Khoruts A., Louie T., Martinelli L.P. (2011). Treating Clostridium difficile Infection with Fecal Microbiota Transplantation. Clin. Gastroenterol. Hepatol..

[113] Aas J., Gessert C.E., Bakken J.S. (2003). Recurrent *Clostridium difficile* Colitis: Case Series Involving 18 Patients Treated with Donor Stool Administered via a Nasogastric Tube. Clin. Infect. Dis..

[114] Youngster I., Sauk J., Pindar C., Wilson R.G., Kaplan J.L., Smith M.B., Alm E.J., Gevers D., Russell G.H., Hohmann E.L. (2014). Fecal Microbiota Transplant for Relapsing Clostridium difficile Infection Using a Frozen Inoculum from Unrelated Donors: A Randomized, Open-Label, Controlled Pilot Study. Clin. Infect. Dis..

[115] Youngster I., Russell G.H., Pindar C., Ziv-Baran T., Sauk J., Hohmann E.L. (2014). Oral, Capsulized, Frozen Fecal Microbiota Transplantation for Relapsing *Clostridium difficile* Infection. JAMA.

[116] Persky S.E., Brandt L.J. (2000). Treatment of recurrent Clostridium difficile-associated diarrhea by administration of donated stool directly through a colonoscope. Am. J. Gastroenterol..

[117] Quraishi M.N., Moakes C.A., Yalchin M., Segal J., Ives N.J., Magill L., Manzoor S.E., Gerasimidis K., Loi S., McMullan C. (2024). Determining the optimal route of faecal microbiota transplant in patients with ulcerative colitis: The STOP-Colitis pilot RCT. Effic. Mech. Eval..

[118] Mendelsohn R.B., Kaltsas A., King S., Hwang C., Kassam Z., Abend A.M., Kramer E., Kamboj M. (2022). Fecal Microbiota Transplantation Is Safe for Clostridiodies difficile Infection in Patients with Solid Tumors Undergoing Chemotherapy. Dig. Dis. Sci..

[119] Hvas C.L., Dahl Jørgensen S.M., Jørgensen S.P., Storgaard M., Lemming L., Hansen M.M., Erikstrup C., Dahlerup J.F. (2019). Fecal Microbiota Transplantation Is Superior to Fidaxomicin for Treatment of Recurrent Clostridium difficile Infection. Gastroenterology.

[120] Sharma A., Roy A., Premkumar M., Verma N., Duseja A., Taneja S., Grover S., Chopra M., Dhiman R.K. (2022). Fecal microbiota transplantation in alcohol-associated acute-on-chronic liver failure: An open-label clinical trial. Hepatol. Int..

[121] Sims M.D., Khanna S., Feuerstadt P., Louie T.J., Kelly C.R., Huang E.S., Hohmann E.L., Wang E.E.L., Oneto C., Cohen S.H. (2023). Safety and Tolerability of SER-109 as an Investigational Microbiome Therapeutic in Adults with Recurrent Clostridioides difficile Infection: A Phase 3, Open-Label, Single-Arm Trial. JAMA Netw. Open.

[122] Haifer C., Paramsothy S., Kaakoush N.O., Saikal A., Ghaly S., Yang T., Luu L.D.W., Borody T.J., Leong R.W. (2022). Lyophilised oral faecal microbiota transplantation for ulcerative colitis (LOTUS): A randomised, double-blind, placebo-controlled trial. Lancet Gastroenterol. Hepatol..

[123] Kao D., Roach B., Silva M., Beck P., Rioux K., Kaplan G.G., Chang H.J., Coward S., Goodman K.J., Xu H. (2017). Effect of oral capsule– vs colonoscopy-delivered fecal microbiota transplantation on recurrent Clostridium difficile infection: A randomized clinical trial. JAMA.

[124] Garey K.W., Dubberke E.R., Guo A., Harvey A., Yang M., García-Horton V., Fillbrunn M., Wang H., Tillotson G.S., Bancke L.L. (2023). Effect of Fecal Microbiota, Live-Jslm (REBYOTA [RBL]) on Health-Related Quality of Life in Patients with Recurrent *Clostridioides difficile* Infection: Results from the PUNCH CD.3 Clinical Trial. Open Forum Infect. Dis..

[125] Lee C., Louie T., Bancke L., Guthmueller B., Harvey A., Feuerstadt P., Khanna S., Orenstein R., Dubberke E.R. (2023). Safety of fecal microbiota, live-jslm (REBYOTA ^TM^) in individuals with recurrent *Clostridioides difficile* infection: Data from five prospective clinical trials. Ther. Adv. Gastroenterol..

[126] Tang G., Yin W., Liu W. (2017). Is frozen fecal microbiota transplantation as effective as fresh fecal microbiota transplantation in patients with recurrent or refractory Clostridium difficile infection: A meta-analysis?. Diagn. Microbiol. Infect. Dis..

[127] Lee C.H., Steiner T., Petrof E.O., Smieja M., Roscoe D., Nematallah A., Weese J.S., Collins S., Moayyedi P., Crowther M. (2016). Frozen vs Fresh Fecal Microbiota Transplantation and Clinical Resolution of Diarrhea in Patients with Recurrent *Clostridium difficile* Infection. JAMA.

[128] Bakker G.J., Nieuwdorp M. (2017). Fecal Microbiota Transplantation: Therapeutic Potential for a Multitude of Diseases beyond Clostridium difficile. Microbiol Spectr..

[129] Alang N., Kelly C.R. (2015). Weight gain after fecal microbiota transplantation. Open Forum Infect. Dis..

[130] Vrieze A., Van Nood E., Holleman F., Salojärvi J., Kootte R.S., Bartelsman J.F.W.M., Dallinga-Thie G.M., Ackermans M.T., Serlie M.J., Oozeer R. (2012). Transfer of intestinal microbiota from lean donors increases insulin sensitivity in individuals with metabolic syndrome. Gastroenterology.

[131] Di Luccia B., Crescenzo R., Mazzoli A., Cigliano L., Venditti P., Walser J.C., Widmer A., Baccigalupi L., Ricca E., Iossa S. (2015). Rescue of fructose-induced metabolic syndrome by antibiotics or faecal transplantation in a rat model of obesity. PLoS ONE.

[132] Sung M.M., Kim T.T., Denou E., Soltys C.L.M., Hamza S.M., Byrne N.J., Masson G., Park H., Wishart D.S., Madsen K.L. (2017). Improved glucose homeostasis in obese mice treated with resveratrol is associated with alterations in the gut microbiome. Diabetes.

[133] Ng S.C., Xu Z., Mak J.W.Y., Yang K., Liu Q., Zuo T., Tang W., Lau L., Lui R.N., Wong S. (2022). Microbiota engraftment after faecal microbiota transplantation in obese subjects with type 2 diabetes: A 24-week, double-blind, randomised controlled trial. Gut.

[134] Chiu C.C., Ching Y.H., Li Y.P., Liu J.Y., Huang Y.T., Huang Y.W., Yang S.S., Huang W.C., Chuang H.L. (2017). Nonalcoholic fatty liver disease is exacerbated in high-fat diet-fed gnotobiotic mice by colonization with the gut microbiota from patients with nonalcoholic steatohepatitis. Nutrients.

[135] Zhou D., Pan Q., Shen F., Cao H.X., Ding W.J., Chen Y.W., Fan J.G. (2017). Total fecal microbiota transplantation alleviates high-fat diet-induced steatohepatitis in mice via beneficial regulation of gut microbiota. Sci. Rep..

[136] Cani P.D., Van Hul M., Lefort C., Depommier C., Rastelli M., Everard A. (2019). Microbial regulation of organismal energy homeostasis. Nat. Metab..

[137] Craven L., Rahman A., Nair Parvathy S., Beaton M., Silverman J., Qumosani K., Hramiak I., Hegele R., Joy T., Meddings J. (2020). Allogenic Fecal Microbiota Transplantation in Patients with Nonalcoholic Fatty Liver Disease Improves Abnormal Small Intestinal Permeability: A Randomized Control Trial. Am. J. Gastroenterol..

[138] Leith D., Lin Y.Y., Brennan P. (2024). Metabolic Dysfunction-associated Steatotic Liver Disease and Type 2 Diabetes: A Deadly Synergy. Touchrev. Endocrinol..

[139] Zheng L., Wen X.L., Duan S.L., Ji Y.Y. (2022). Fecal microbiota transplantation in the metabolic diseases: Current status and perspectives. World J. Gastroenterol..

[140] Witjes J.J., Smits L.P., Pekmez C.T., Prodan A., Meijnikman A.S., Troelstra M.A., Bouter K.E.C., Herrema H., Levin E., Holleboom A.G. (2020). Donor Fecal Microbiota Transplantation Alters Gut Microbiota and Metabolites in Obese Individuals with Steatohepatitis. Hepatol. Commun..

[141] Lee G., You H.J., Bajaj J.S., Joo S.K., Yu J., Park S., Kang H., Park J.H., Kim J.H., Lee D.H. (2020). Distinct signatures of gut microbiome and metabolites associated with significant fibrosis in non-obese NAFLD. Nat. Commun..

[142] Stols-Gonçalves D., Mak A.L., Madsen M.S., van der Vossen E.W.J., Bruinstroop E., Henneman P., Mol F., Scheithauer T.P.M., Smits L., Witjes J. (2023). Faecal Microbiota transplantation affects liver DNA methylation in Non-alcoholic fatty liver disease: A multi-omics approach. Gut Microbes.

[143] Bajaj J.S., Kassam Z., Fagan A., Gavis E.A., Liu E., Cox I.J., Kheradman R., Heuman D., Wang J., Gurry T. (2017). Fecal microbiota transplant from a rational stool donor improves hepatic encephalopathy: A randomized clinical trial. Hepatology.

[144] Høyer K.L., Dahl Baunwall S.M., Kornum D.S., Klinge M.W., Drewes A.M., Yderstræde K.B., Thingholm L.B., Mortensen M.S., Mikkelsen S., Erikstrup C. (2025). Faecal microbiota transplantation for patients with diabetes type 1 and severe gastrointestinal neuropathy (FADIGAS): A randomised, double-blinded, placebo-controlled trial. eClinicalMedicine.

[145] Costello S.P., Hughes P.A., Waters O., Bryant R.V., Vincent A.D., Blatchford P., Katsikeros R., Makanyanga J., Campaniello M.A., Mavrangelos C. (2019). Effect of Fecal Microbiota Transplantation on 8-Week Remission in Patients with Ulcerative Colitis: A Randomized Clinical Trial. JAMA.

[146] Chen Q., Fan Y., Zhang B., Yan C., Zhang Q., Ke Y., Chen Z., Wang L., Shi H., Hu Y. (2023). Capsulized Fecal Microbiota Transplantation Induces Remission in Patients with Ulcerative Colitis by Gut Microbial Colonization and Metabolite Regulation. Microbiol. Spectr..

[147] Ding X., Li Q., Li P., Zhang T., Cui B., Ji G., Lu X., Zhang F. (2019). Long-Term Safety and Efficacy of Fecal Microbiota Transplant in Active Ulcerative Colitis. Drug Saf..

[148] Fang H., Fu L., Li X., Lu C., Su Y., Xiong K., Zhang L. (2021). Long-term efficacy and safety of monotherapy with a single fresh fecal microbiota transplant for recurrent active ulcerative colitis: A prospective randomized pilot study. Microb. Cell Factories.

[149] Holvoet T., Joossens M., Vázquez-Castellanos J.F., Christiaens E., Heyerick L., Boelens J., Verhasselt B., van Vlierberghe H., De Vos M., Raes J. (2021). Fecal Microbiota Transplantation Reduces Symptoms in Some Patients with Irritable Bowel Syndrome with Predominant Abdominal Bloating: Short- and Long-term Results from a Placebo-Controlled Randomized Trial. Gastroenterology.

[150] Johnsen P.H., Hilpüsch F., Cavanagh J.P., Leikanger I.S., Kolstad C., Valle P.C., Goll R. (2018). Faecal microbiota transplantation versus placebo for moderate-to-severe irritable bowel syndrome: A double-blind, randomised, placebo-controlled, parallel-group, single-centre trial. Lancet Gastroenterol. Hepatol..

[151] Madsen A.M.A., Halkjær S.I., Christensen A.H., Günther S., Browne P.D., Kallemose T., Hansen L.H., Petersen A.M. (2021). The effect of faecal microbiota transplantation on abdominal pain, stool frequency, and stool form in patients with moderate-to-severe irritable bowel syndrome: Results from a randomised, double-blind, placebo-controlled study. Scand. J. Gastroenterol..

[152] Mocanu V., Zhang Z., Deehan E.C., Kao D.H., Hotte N., Karmali S., Birch D.W., Samarasinghe K.K., Walter J., Madsen K.L. (2021). Fecal microbial transplantation and fiber supplementation in patients with severe obesity and metabolic syndrome: A randomized double-blind, placebo-controlled phase 2 trial. Nat. Med..

[153] Leong K.S.W., Jayasinghe T.N., Wilson B.C., Derraik J.G.B., Albert B.B., Chiavaroli V., Svirskis D.M., Beck K.L., Conlon C.A., Jiang Y. (2020). Effects of Fecal Microbiome Transfer in Adolescents with Obesity: The Gut Bugs Randomized Controlled Trial. JAMA Netw. Open.

